# Optimizing Atrial Fibrillation Care: Comparative Assessment of Anticoagulant Therapies and Risk Factors

**DOI:** 10.3390/clinpract14010027

**Published:** 2024-02-12

**Authors:** Marius Rus, Adriana Ioana Ardelean, Simina Crisan, Paula Marian, Oana Lilliana Pobirci, Veronica Huplea, Claudia Judea Pusta, Gheorghe Adrian Osiceanu, Claudia Elena Stanis, Felicia Liana Andronie-Cioara

**Affiliations:** 1 Department of Medical Disciplines, Faculty of Medicine and Pharmacy, University of Oradea, 410073 Oradea, Romania; rusmarius@uoradea.ro (M.R.); paula.marian85@gmail.com (P.M.); 2 Department of Preclinical Disciplines, Faculty of Medicine and Pharmacy, University of Oradea, 410073 Oradea, Romania; adrianaardelean@uoradea.ro; 3 Cardiology Department, “Victor Babes” University of Medicine and Pharmacy, 2 Eftimie Murgu Sq., 300041 Timisoara, Romania; urseanusimina@yahoo.com; 4 Institute of Cardiovascular Diseases Timisoara, 13A Gheorghe Adam Street, 300310 Timisoara, Romania; 5 Research Center of the Institute of Cardiovascular Diseases Timisoara, 13A Gheorghe Adam Street, 300310 Timisoara, Romania; 6 Department of Psycho Neuroscience and Recovery, Faculty of Medicine and Pharmacy, University of Oradea, 410073 Oradea, Romania; oanap_30@yahoo.com (O.L.P.); hupleaveronica@yahoo.com (V.H.); fcioara@uoradea.ro (F.L.A.-C.); 7 Department of Morphological Disciplines, Faculty of Medicine and Pharmacy, University of Oradea, 410073 Oradea, Romania; cjudeapusta@uoradea.ro (C.J.P.); osiceanuadrian@yahoo.com (G.A.O.); 8 Faculty of Medicine and Pharmacy, University of Oradea, 410073 Oradea, Romania

**Keywords:** atrial fibrillation, risk factors, thromboembolic events, novel oral anticoagulants, complications, hospitalization, medication monitoring

## Abstract

Background and objectives: Atrial fibrillation (AF) is a common arrhythmia associated with various risk factors and significant morbidity and mortality. Materials and methods: This article presents findings from a study involving 345 patients with permanent AF. This study examined demographics, risk factors, associated pathologies, complications, and anticoagulant therapy over the course of a year. Results: The results showed a slight predominance of AF in males (55%), with the highest incidence in individuals aged 75 and older (49%). Common risk factors included arterial hypertension (54%), dyslipidemia, diabetes mellitus type 2 (19.13%), and obesity (15.65%). Comorbidities such as congestive heart failure (35.6%), mitral valve regurgitation (60%), and dilated cardiomyopathy (32%) were prevalent among the patients. Major complications included congestive heart failure (32%), stroke (17%), and myocardial infarction (5%). Thromboembolic and bleeding risk assessment using CHA2DS2-VASc and HAS-BLED scores demonstrated a high thromboembolic risk in all patients. The majority of patients were receiving novel oral anticoagulants (NOACs) before admission (73%), while NOACs were also the most prescribed antithrombotic therapy at discharge (61%). Conclusions: This study highlights the importance of risk factor management and appropriate anticoagulant therapy in patients with AF, to reduce complications and improve outcomes. The results support the importance of tailored therapeutic schemes, for optimal care of patients with AF.

## 1. Introduction

Atrial fibrillation is the most common sustained arrhythmia encountered and managed in clinical practice [1]. A supraventricular tachycardia, AF is characterized by disorganized, fast, and irregular atrial electrical activation, as well as an irregular ventricular response [2]. Affecting both cardiac patients, as well as individuals with no history of cardiovascular disease, the prevalence of AF varies from 0.5 to 1% in the general population, to a 10-fold greater value in those aged over 65 [3]. The causes of AF are relatively well defined. Acute hyperthyroidism, vagotonic episodes, or alcohol intoxication may trigger the appearance of paroxysmal AF, while the critical phase after major vascular, abdominal, or thoracic surgery is linked to acute episodes of AF [3]. Additional triggers for AF include other arrhythmias (i.e., atrioventricular nodal re-entry tachycardia—AVNRT); the progression of structural valvular, myocardial, or coronary disease; hypoxia; electrolyte imbalance or metabolic disorders; pericarditis; myocarditis; atrial or conduction tissue degeneration associated with aging; genetical predisposition; male sex; and so forth [4]. Eliminating the modifiable risk factors may prevent AF recurrence.

Based on the duration of progression, AF is classified as follows: first diagnosed—undiagnosed before, irrespective of its duration or the presence/severity of AF-related symptoms; paroxysmal—terminates spontaneously or with intervention within 7 days of onset; persistent—continuously sustained beyond 7 days (including episodes terminated through cardioversion after ≥7 days); long-standing persistent—continuous for >12 months; permanent—accepted by the patient and physician, with no further attempts to restore or maintain sinus rhythm [4].

AF symptoms greatly vary, ranging from completely asymptomatic or silent to hemodynamically unstable patients. Most commonly, patients present mild-to-severe palpitations. The hemodynamic effect can be extremely pronounced, based on the atrial quiver and the ventricular response, with severe symptomatic hypotension, pulmonary edema, or angina in some cases [3,4,5]. The evaluation of a patient with AF should include a detailed history and a thorough physical examination. Defining the clinical type of AF, as well as identifying any reversible causes (e.g., hyperthyroidism or anemia), are crucial components in the management of AF [2,6].

The clinical importance of AF is determined by the loss of atrial contraction, an inadequately increased ventricular response, and the absence of atrial auricular contraction. As a result, the blood flow pools, thus increasing the risk of local clot formation and thromboembolic events [3].

AF is an important morbidity and mortality factor, as it is linked to an increased risk of stroke, heart failure, and even death. Patients’ quality of life is impaired in more than 60% of cases, due to the burden, comorbidities, as well as psychological factors and medication. Repeated hospitalizations, ranging from a 10 to 40% annual rate, and prevalent depression (16–20%) further contribute to a decreased quality of life [1,2,3,4]. Up to 20–30% of all ischemic strokes, and 10% of cryptogenic strokes, are AF-related, while the risk of heart failure also rises to 20–30% due to the excessive ventricular rate and irregular ventricular contractions [3]. All these enhanced risk factors contribute to an excessive mortality rate with a 1.5–3.5-fold increase, in comparison to healthy individuals [4].

To decrease the risk of mortality, stroke, and hospitalization associated with AF, anticoagulants are pivotal in the management of this dysrhythmia, due to its high occurrence of thromboembolic events. Until the past decade, the most commonly used anticoagulant therapy consisted of vitamin K antagonists (VKAs)—warfarin and acenocoumarol. However, due to their large interpatient variability in the slow onset and offset of action, extensive food and drug interactions, need for coagulation monitoring and dose adjustments, as well as dose response, VKAs’ use created a complicated management scheme [7]. Thus, their eventual limitation was inevitable with the appearance of the novel oral anticoagulants (NOACs) [5]. Four NOACs are currently available for the prevention of thromboembolic events in patients with AF: apixaban, edoxaban, rivaroxaban, and dabigatran. The formers are oral direct Factor Xa inhibitors, while the latter is an oral direct thrombin inhibitor [7].

We investigated management strategies and modifiable risk factors associated with atrial fibrillation, with the goal of enhancing treatment outcomes, reducing complications, and improving patient care. Furthermore, special attention was given when assessing the comparative efficacy and safety of different anticoagulation therapies (e.g., NOACs vs. VKAs) for stroke prevention in patients with AF. This study aims to contribute to the existing knowledge base, guide clinical decision making, and ultimately improve patient outcomes in the management of this common cardiac arrhythmia.

## 2. Materials and Methods

In this paper, we study 345 symptomatic patients, diagnosed with permanent AF, and perform a systematic analysis of different clinical and paraclinical characteristics. This study is retrospective, taking place over the course of one year (March 2022–March 2023). This study was approved by the ethics committee of Bihor County Clinical Emergency Hospital.

The included patients were diagnosed with permanent AF with a moderate heart rate, several of them presenting multiple hospitalizations during the years, with all of them being unsuccessful in re-establishing sinus rhythm. The persistence of AF creates a higher risk of thromboembolic complications, with some of the patients’ histories already presenting thromboembolic events.

The exclusion criteria were as follows: first-diagnosed, paroxysmal, or long-standing persistent AF, patients with AF re-established to sinus rhythm, permanent AF with a fast heart rate.

The diagnosis of permanent AF was established based on the general physical examination and the cardiovascular exam: palpitations, dyspnea, vertigo, syncope, fatigue, chest pain, and arrhythmic cardiac sounds, asynchronous with the pulse. The EKG confirmed the diagnosis.

Baseline characteristics of the study population were systematically analyzed using data obtained from observational charts (Table 1). The parameters we followed were the age group, sex, number of hospitalization days, AF risk factors and associated pathologies, AF complications, anticoagulant therapy before admission, anticoagulant therapy during hospitalization, antithrombotic therapy prescribed at discharge, and medication monitoring.

A statistical analysis was performed with STATISTICA 8.0, using the chi-test and z–test for 2 proportions and one-way ANOVA test for comparing multiple categorical variables. A *p*-value ≤ 0.05 was considered to be statistically significant.

As risk stratification tools, and according to the European Society of Cardiology (ESC) guidelines, we used the CHA2DS2-VASc score to estimate the risk of stroke in patients with AF, as well as the HAS-BLED score to assess the risk of bleeding complications. These risk stratification scores identify patients who may require closer monitoring, or adjustments in their antithrombotic medication management.

The CHA2DS2-VASc score considers various clinical risk factors associated with stroke, and assigns a score to each factor, summing up the points assigned to each parameter (Table 2). The total score can range from 0 to 9, a higher score indicating a higher risk of stroke. Congestive heart failure/left ventricular (LV) dysfunction is defined as an LV ejection fraction ≤40%. Hypertension includes patients with current antihypertensive medication. Vascular disease refers to prior myocardial infarction, peripheral artery disease, or aortic plaques.

The calculation of the HAS-BLED score uses a similar method to the CHA2DS2-VASc score, involving different parameters (Table 3). Each parameter carries a score of 1, resulting in a total score ranging from 0 to 9. A higher score indicates an increased risk of bleeding complications.

## 3. Results

**Figure 1:**
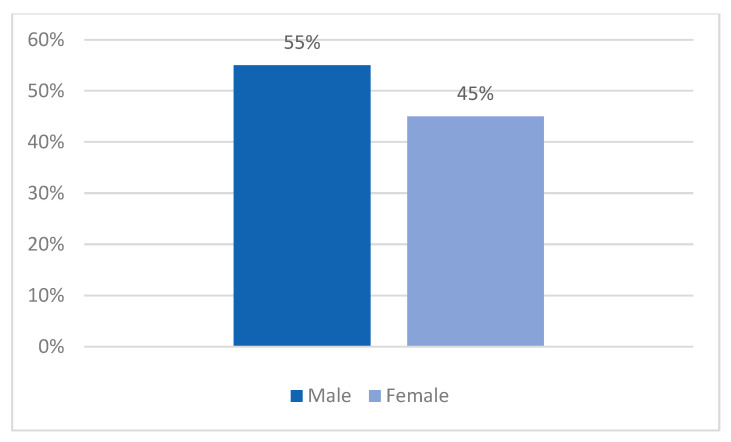
Sex distribution.

In this paper, 55% of subjects were male with 45% being female, emphasizing a slight prevalence of the masculine sex (*p* = 0.35) (Figure 1).

**Figure 2:**
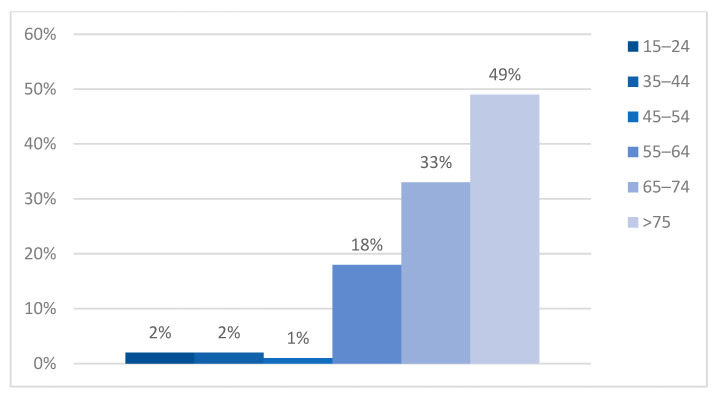
Age distribution.

Most cases in this study were included in the ‘75 and over’ age category (168 patients, 49%). In 33% of cases, the patients were part of the 65–74 age group, while 18% of them pertained to the 55–64 group (*p* = 0.001). The incidence of atrial fibrillation significantly decreased along with age, with data supported by the small number of younger patients included in this study—2% in the 15–24 y.o. group, 2% in the 35–44 group, and 1% in the 45–54 age group (*p* = 0.8) (Figure 2).

**Figure 3:**
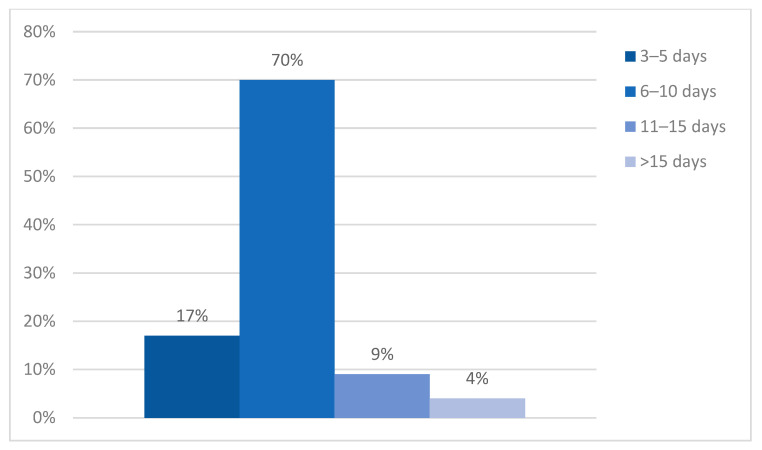
Hospitalization duration.

Due to the characteristics of patients with atrial fibrillation, e.g., older age and several associated morbidities, which require thorough investigations and prolonged supervision, 70% of subjects were admitted for 6 to 10 days. Such a prolonged period, of course, implied higher hospitalization costs. On that same note, 9% of study subjects were admitted for more than 10 days, with 4% spending more than 2 weeks (15 days on average) in the hospital. The shortest admission period, seen in 17% of cases, was 3 to 5 days (*p* ˂ 0.0001) (Figure 3).

**Figure 4:**
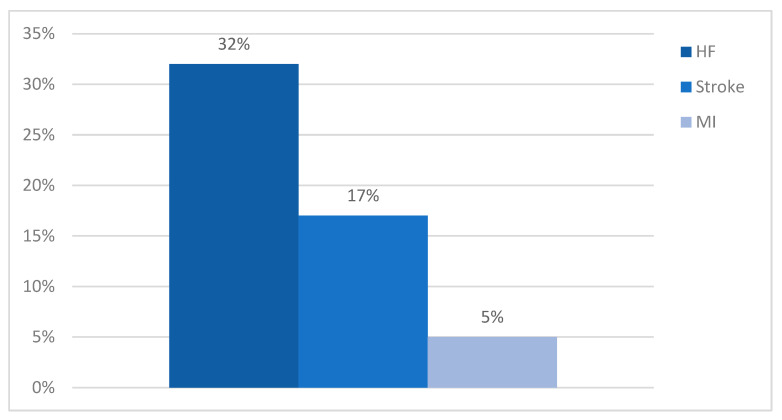
Complications.

Complications related to atrial fibrillation were present in 186 patients. Our study highlighted congestive heart failure as a major complication in patients with AF (32%), followed closely by stroke in almost 17% of cases. In 5% of cases, the patients suffered a myocardial infarction (Figure 4).

**Figure 5:**
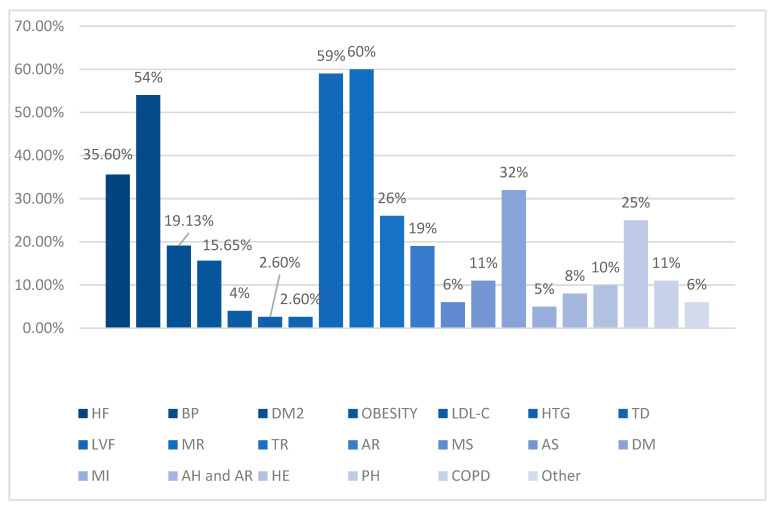
Comorbidities.

In this study, among the major risk factors, 16% of patients suffered a stroke, with 49% of the patients being above the age of 75. Although considered minor factors, congestive heart failure (HF) (35.6%) and arterial hypertension (54%) were relatively common (*p* = 0.02) (Figure 5).

Other than arterial hypertension (BP) (54%), diabetes mellitus type 2 (DM2) (19.13%) and obesity (15.65%) were relatively frequent as well (*p* ˂ 0.0001).

Minor risk contributors were hypercholesterolemia (LDL-C) (4%), hypertriglyceridemia (TG) (2.6%), and thyroid dysfunction (TD) (2.6%).

A multitude of associated pathologies were found in patients with atrial fibrillation, each comorbidity further increasing the risk of complications and mortality. Left ventricular failure (LVF) was a major associate, present in more than 59% of cases, followed by arterial hypertension (54%) and congestive heart failure (35.6%) (*p* = 0.28). However, mitral valve regurgitation (MR) (grade 2 to 4) represented the most frequent comorbidity, seen in 60% of cases, while 26% of patients presented tricuspid regurgitation (TR) and 19% presented aortic regurgitation (AR). Mitral or aortic stenosis (MS and AS) held a lower position, with 6% and 11% of patients diagnosed with these pathologies. Dilated cardiomyopathy (DM) affected 32% of study subjects, while only 5% of them suffered a myocardial infarction (MI) (p ˂ 0.0001).

A labile INR was found in 30% of cases (*p* = 0.0001), while 8% of patients presented abnormal renal and/or hepatic function (AH and AR) (*p* ˂ 0.001). Both of these factors are modifiable through medication. Among the unmodifiable risk factors, 96% of patients were 45 years old or older (*p* ˂ 0.001) and 16% suffered a stroke (*p* ˂ 0.001), while 10% experienced a hemorrhagic event (HE) (*p* ˂ 0.0001) (Figure 5).

Heart diseases were not the only pathologies found in the participants of this study. Pulmonary comorbidities were present in a relatively high proportion, with pulmonary hypertension (PH) present in 25% of cases and chronic obstructive pulmonary disease (COPD) in 11% of cases (*p* < 0.00001). Other respiratory comorbidities (pleural effusion, pneumonia, asthma) were found in 6% of cases (*p* < 0.00001).

**Figure 6:**
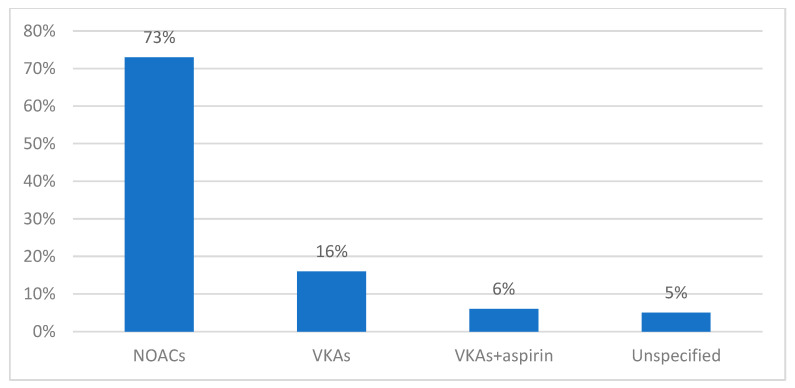
Antithrombotic medication before admission.

The most frequently used antithrombotic medication before admission to the hospital was NOACs (apixaban, edoxaban, rivaroxaban, dabigatran), with 73% of patients using these drugs in monotherapy. In total, 16% of patients were on VKAs (acenocumarol or warfarin), while 6% had been previously prescribed VKAs and aspirin together (Figure 6).

**Figure 7:**
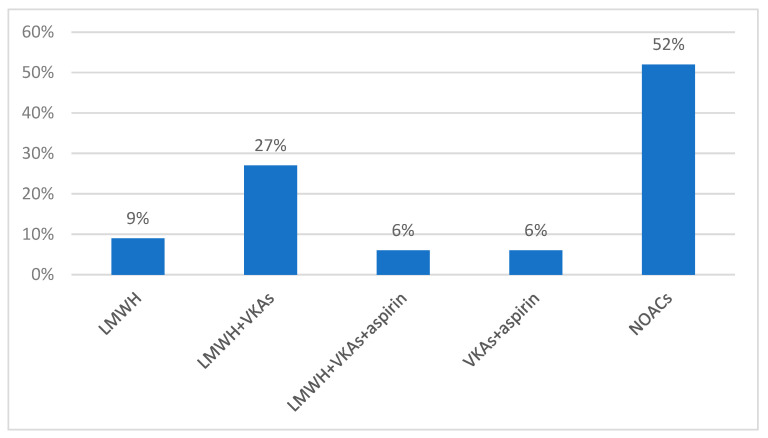
Antithrombotic medication during hospitalization.

During hospitalization, when urgent treatment was needed, low-molecular-weight heparin (LMWH) was used in monotherapy in 9% of cases. LMWH and VKAs were used together in 27% of patients, with a loading dose of acenocumarol for 2 to 3 days and double the maintenance dose (4–6 mg), followed by maintenance treatment of 2 mg/day. LMWH was interrupted when the INR reached a therapeutic value for 2 consecutive days. In total, 6% of patients were given a combination of LMWH, VKAs, and aspirin, and another 6% used acenocumarol and aspirin together (p ˂ 0.001). Up to 52% of patients were given a NOAC during admission (*p* = 0.70) (Figure 7).

**Figure 8:**
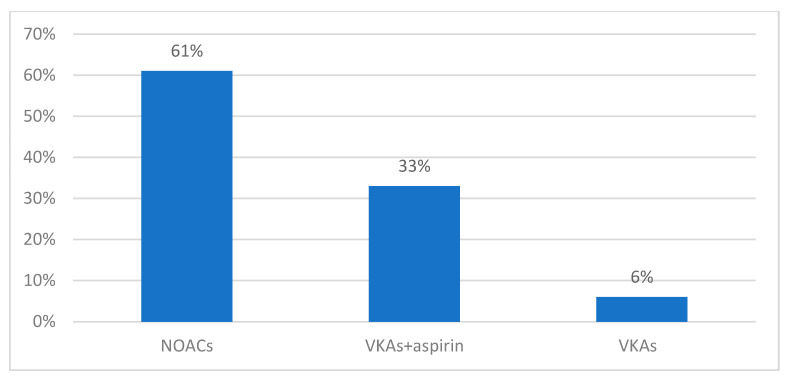
Antithrombotic therapy prescribed at discharge.

At discharge, 61% of patients were prescribed a NOAC in monotherapy, with 33% receiving VKAs (acenocumarol) and aspirin together (*p* = 0.002). INR monitoring was required once every two weeks. Acenocumarol as a single drug was only prescribed in 6% of cases (Figure 8).

## 4. Discussion

According to recent studies, it is currently greatly recognized that the epidemiology of AF differs between men and women [8]. A slight prevalence of the male sex is supported by several cohort findings, including the ARIC (Atherosclerosis Risk in Communities) study, which notes a higher lifetime risk of atrial fibrillation in White men compared to White women [9]. This small predominance can be attributed to sex-related risk factors, and life and work conditions that predispose men to cardiovascular diseases, including atrial fibrillation. However, according to a different study, as age increases, the gap between the sexes also decreases. Over the age of 75, the prevalence of atrial fibrillation seems to be higher in women. This finding could possibly be attributed to their increased longevity [10,11]. Thus, due to existing conflicting data between works, it is extremely difficult to affirm whether sex plays a decisive role in the development of atrial fibrillation [12,13].

Additionally, the prevalence of AF varies among different ethnic populations. Over the course of years, numerous epidemiological studies consistently found a gradual rise in the incidence and prevalence of AF with advancing age [13]. However, an overwhelming majority of this study’s participants were above the age of 45 years old. Thus, we might argue that the influence of age over the epidemiology of AF might be overruled by the presence of significant risk factors, despite a relatively young age.

Similar to other studies, our work emphasizes that not only have the hospitalization rates for AF increased exponentially, but also the cost of hospitalization, despite the overall decline in hospital mortality [14]. This arrhythmia increases the risk of overall mortality, but also the morbidity resulting from complications, such as stroke, heart failure, and impaired quality of life. Such severe complications could be caused by an untreated or undertreated AF, in terms of both antiarrhythmic therapies, as well as antithrombotic medication. Thus, the economic burden associated with AF is on a constant rise [15].

Similar to the findings of other papers, this work highlights the higher prevalence and incidence of a multitude of comorbidities patients with AF present, compared to healthy individuals [16]. Mitral valvular pathology, left ventricular failure, dilated cardiomyopathy, hypertension, and heart failure were the conditions largely associated with AF in this work. These comorbidities could also possibly explain the origins of AF in some of the cases. However, when it comes to mortality, other studies argue that AF does not have an augmented impact, despite the greater comorbidity burden [16].

Risk factor management is a crucial element in optimizing AF care. The focus falls on identifying the modifiable risk factors. Once established, these factors could be influenced, thus contributing to decreasing the risk of further complications and stalling the evolution of the disease.

Numerous guidelines (ESC, American Heart Association—AHA, Asian Pacific Heart Rhythm Society—APHRS) have reiterated the importance of an integrated holistic approach of patients with AF across healthcare levels and among different specialties [4,17,18]. Whether referring to the ESC Atrial Fibrillation Better Care pathway (A—anticoagulation/avoid stroke, B—better symptom management, C—cardiovascular and comorbidity optimization), or the AHA SOS pathway (stroke risk assessment and treatment, optimizing all modifiable risk factors, and symptom management), no doubt remains regarding the superiority of such approaches. As shown by several studies, such as the GLORIA-AF registry or the ENGAGE AF-TIMI 48 trial, compliance with the ABC/SOS streamline is associated with a reduced risk of major adverse events (including mortality, thromboembolism, and major cardiovascular events), thus improving the clinical outcomes of patients with AF [19,20,21,22]. Moreover, both streamlines have been associated with significant reduction in the risk of all-cause death, composite outcomes, as well as health-related costs [4,17].

The 2021 APHRS consensus notes the unaddressed questions and remaining gaps in the management of AF, especially referring to patients with COVID-19 [18]. Simultaneously, it underscores the pivotal role of the physician’s decision making in navigating the approach of this disease [18].

Despite the abundance of evidence and numerous studies advocating for the efficacy of the ABC/SOS pathway in enhancing patient outcomes, this facet of AF may be frequently overlooked and underused. As a result, the morbidity and mortality of AF remain increased, despite continual advancements in medical care.

One of the modifiable risk factors is represented by high blood pressure. The hemodynamic stress caused by arterial hypertension leads to a surge in the intra-atrial pressure [23]. This constant pressure increase causes structural damage of the heart tissue over time, as well as electrical changes in the atrial conduction system [24]. Thus, such a frequently encountered risk factor in the general population predisposes to the occurrence of atrial fibrillation. Several studies have discussed the importance of poorly controlled blood pressure, due to its association with an elevated risk of AF [23,24]. Clinical trial data also indicate a linear association between blood pressure management and adverse cardiovascular outcomes, suggesting a correlation between lower blood pressure levels and better cardiovascular results [25]. In conclusion, there is a consensus that managing blood pressure represents a viable strategy for reducing the risk in individuals with atrial fibrillation (AF) [26,27].

Mitral valve regurgitation emerges as a significant risk factor identified in our study. Presently, there exists ambiguity regarding the optimal sequencing of treatment interventions between valvular disease and arrhythmia. It remains uncertain whether prioritizing the management of valvular disease precedes addressing the arrhythmia, or vice versa. *Kim* et al. studied the association between moderately severe MR and AF, in the quest to find the answer to this clinical dilemma. The conclusion was that, in order to completely solve the issue, both pathologies should be addressed in order to decrease the risk of uniform progression [28,29].

In numerous clinical studies, considerable emphasis has been placed on heart failure (HF) as both a risk factor and a complication of atrial fibrillation (AF) [30,31,32]. The pathological changes observed in HF lead to a proarrhythmic terrain due to sinus node dysfunction, favoring the appearance and maintenance of AF [33]. Atrial fibrillation can be the primary cause for the development of HF, as well as the stimulus for HF decompensation [33]. Most often, it is difficult to distinguish which entity occurred first, due to their shared risk factors. Many studies have focused on establishing effective therapeutic schemes. However, optimal treatment strategies for patients with HF-AF remain unclear [33].

Obesity is a potent risk factor for AF, as consistently demonstrated by epidemiological studies [34]. There are numerous theories on why AF can develop in patients with obesity, especially in patients with metabolic syndrome. The diastolic dysfunction of the left ventricle, electrical and structural remodeling of the atria, local inflammation of the atria, as well as atrial irritability due to epicardial and pericardiac adipose tissue represent a few of these hypotheses [34]. Several trials have demonstrated that to achieve a significant decrease in AF risk, a larger sustained weight loss is necessary (at least 10% of body weight) [34,35]. The official guidelines (ESC, AHA) also emphasize the significance of weight loss in improving the outcome of rhythm control in patients with AF [4,17].

Myocardial infarction can also be an aggravating factor of AF, due to the remodeling of the atria occurring after atrial ischemia, as well as a complication of AF itself [36]. Other works discuss the common risk factors between coronary artery disease and AF [37].

Other conditions associated with AF include respiratory disorders, such as pulmonary hypertension (PH) or chronic obstructive pulmonary disease (COPD). Pulmonary hypertension, defined as a mean pulmonary artery pressure ≥25 mmHg at rest or ≥30 mmHg with exercise, induces increased pressure and volume overload in the right heart [17,38]. Similarly, COPD, a primary cause of mortality and morbidity worldwide, has been independently linked to AF, although the precise pathophysiological mechanisms remain incompletely understood [17,39]. Factors such as hypoxia, hypercapnia, diastolic dysfunction, oxidative stress, and inflammation may instigate structural and electrophysiological arrhythmogenic changes [38,39]. Consequently, supraventricular arrhythmias manifest more frequently in patients with advanced respiratory disorders compared to ventricular arrhythmias [38]. Recent investigations suggest that the presence of these respiratory conditions independently impairs the prognosis, influences AF progression, alters the success rate of cardioversion, and predisposes to AF recurrence following catheter ablation [38,39]. Moreover, the heightened risk of bleeding and hemoptysis necessitates careful consideration in anticoagulated patients with AF and respiratory comorbidities [38]. Collectively, these factors contribute to elevated cardiovascular and all-cause mortality rates.

Even though not all studies show significant relation between the extensive list of pathologies we detected and AF [40], multiple large-population-based works have emphasized the increased risk of developing AF in patients presenting any of the aforementioned elements, and the importance of their proper management as part of an AF optimal treatment scheme [4,17,18,19,20,21,22,23,41,42].

Regarding complications, the international normalized ratio (INR) was created to express the coagulation state, with several formulas elaborated on to assess the quality of anticoagulation [43]. Thus, a labile INR (outside of the 2–3 range) stands for poor anticoagulant control, increasing the risk of stroke or bleeding events. The time in the therapeutic range (TTR) is one of the formulas proposed to assess the level of anticoagulation in patients prescribed VKAs [43]. The FANTASIIA study, with a total of 1290 patients recruited, reported that patients with AF spend less than half the time within the therapeutic range when treated with VKAs [43]. Our study emphasizes the importance of a labile INR as a risk factor for AF-related complications, as well as the ease of use of NOACs compared to VKAs. However, the anticoagulant status is influenced not only by the prescribed antithrombotic therapy, but also by underlying conditions, such as liver or renal disease. No matter the case, keeping the INR within the accepted range in anticoagulated patients is one of the pillars of proper AF management.

Stroke, as a complication of AF, has been widely discussed along the years [44,45]. It has been observed that patients with recurring ischemic stroke display evidence of undiagnosed and untreated AF. Long-term monitoring of cardiac function (e.g., Holter) is essential in detecting this arrhythmia. Wańkowicz et al. highlight common recognized risk factors that AF and ischemic stroke share [46]. Attending to these common modifiable elements individually might reduce the risk of both afflictions [47]. Although anticoagulation alone might not decrease the risk of stroke in patients with AF, Evans et al. suggest that the morbidity due to antithrombotic medication is by far exceeded by the number of preventable strokes [48]. Moreover, another study identified independent associations between major bleeding events and stroke-specific factors in anticoagulated patients with stroke and AF [49]. However, according to the PREFER in AF study, the initiation of antithrombotic medication should not be delayed or prevented, even despite elevated bleeding risk scores [47]. All in all, individual characteristics of patients with stroke and AF should be considered in secondary prevention, as part of a comprehensive approach [49].

We calculated the clinical-risk-factor-based CHA2DS2-VASc and HAS-BLED scores for all patients, in accordance with the ESC guidelines. When considering the appropriate anticoagulant therapy, we appreciated the thromboembolic risk of each individual, as well as the bleeding risk.

The ESC guidelines have recommended the use of the CHA2DS2-VASc score since 2010, as a class I recommendation for risk stratification in patients with AF. An overlap of risk factors can be found between the CHA2DS2-VASc and the HAS-BLED score. Nevertheless, there are works that prove that the HAS-BLED score performed better than the CHA2DS2-VASc score in anticoagulated patients with AF [50]. While other bleeding scores have been suggested, such as ORBIT or ATRIA, the HAS-BLED score has been proven as superior to both scores in the “real-world” of oral anticoagulated patients with AF [51,52].

It is also important to mention the integrated GARFIELD-AF risk tool and its clinical implications when compared to both HAS-BLED as well as CHA2DS2-VASc [53]. As opposed to using two separate scores to assess the risk of stroke and bleeding, the GARFIELD-AF incorporates the risk of mortality, stroke, and bleeding, in a single calculation [53]. Thus, according to the original study, it allows the physicians to balance the considerations between risks and benefits. Moreover, this score provides information on the impact of NOAC vs. VKA therapy, while also emphasizing the importance of comprehensive secondary prevention [53]. The low-risk patients are also considered, as opposed to CHA2DS2-VASc and HAS-BLED [53]. In clinical practice, the simple HAS-BLED score outperformed the algorithm-based GARFIELD-AF bleeding score regarding major bleedings and clinically relevant non-major (CRNM) bleedings [54].

However, undergoing studies argue that the HAS-BLED score has been developed for patients prescribed warfarin, thus making it much less reliable compared to the CHA2DS2-VASc score, which is well established in both European as well as American guidelines. Since an overwhelming majority of physicians prefer the use of NOACs compared to VKAs, the need to develop a new bleeding score, specifically targeted to the direct-acting oral anticoagulants, has risen [55]. The direct-acting oral anticoagulant (DOAC) score has been developed with the NOAC-treated patients’ characteristics in mind, considering crucial variables such as age, kidney function, and concomitant high-bleeding-risk medication use [55]. So far, the DOAC score seems to outperform HAS-BLED regarding patient outcomes, and an enhanced safety and efficacy profile [56]. Its adoption by physicians worldwide depends on its endorsement by atrial fibrillation official guidelines [55].

According to the ESC 2020 AF guidelines, antithrombotic medication is recommended for all patients with a CHA2DS2-VASc score ≥ 2 for males, and ≥3 for females. If the HAS-BLED score ≥ 3, the modifiable bleeding risk factors should be addressed, but a high bleeding risk score should not be used as a reason to withhold anticoagulant therapy [4]. Based on the CHA2DS2-VASc score, no antiplatelet or anticoagulant therapy should be initiated if no risk factors are present (score 0 in males, score 1 in females). However, all the patients evaluated in this study belong to the high-thromboembolic-risk category, with a minimum score value of 2.

The clearly preferential use of NOACs outside the hospital, and before, during, and after hospitalization, has risen exponentially since their discovery, multiple studies and clinical trials proving they are superior to warfarin for the prevention of stroke and systemic embolism in patients with AF. The significant reduction in intracranial hemorrhage results in a significantly lower mortality [56]. However, the correlation between NOACs and gastrointestinal bleeding continues to remain controversial, with some trials (ARISTOTLE, J-ROCKET AF, ENGAGE TIMI AF 48, RE-LY) inclining toward significant heterogeneity and non-significant bleeding with certain NOACs (lower-dose edoxaban, lower-dose dabigatran), while others (ROCKET AF) clearly indicate more gastrointestinal bleeding with a higher dose of dabigatran [21,57,58,59,60,61]. Whether or not to prescribe NOACs depends on each clinician, as well as the particularities of each patient. However, the evidence suggesting a favorable long-term outcome for the usage of NOACs, compared to warfarin, in patients with AF cannot be completely disregarded.

## 5. Conclusions

This study provides valuable insights into the management and risk factors associated with atrial fibrillation (AF). The findings underscore the importance of identifying and addressing risk factors to prevent complications and improve patient outcomes. Anticoagulant therapy, including VKAs and NOACs, plays a vital role in stroke prevention in patients with AF. The selection of anticoagulant therapy should be tailored to individual patient factors, considering the benefits and risks associated with each option.

This study confirmed that AF is more prevalent in males, particularly in older age groups. Mitral regurgitation was the most common risk factor, followed by left ventricular failure, high blood pressure, congestive heart failure, dyslipidemia, diabetes mellitus type 2, and obesity. These modifiable risk factors should be targeted through lifestyle modifications and appropriate medical interventions to reduce the incidence and progression of AF. Comprehensive management of these associated pathologies is crucial in the overall care of patients with AF.

This study highlights the significant complications associated with untreated or undertreated AF, including heart failure, stroke, and myocardial infarction. This emphasizes the need for timely and effective treatment strategies to control AF and minimize its impact on patient health.

Thromboembolic risk assessment using the CHA2DS2-VASc score demonstrated a high thromboembolic risk in all patients, reinforcing the importance of anticoagulant therapy. After determining the HAS-BLED score, novel oral anticoagulants (NOACs) emerged as the preferred choice due to their ease of use, predictable pharmacokinetics, and reduced risk of interactions compared to traditional vitamin K antagonists.

In conclusion, this study emphasizes the significance of risk factor management and appropriate anticoagulant therapy in patients with AF. By addressing modifiable risk factors, optimizing comorbidity management, and implementing appropriate antithrombotic strategies, healthcare professionals can reduce complications, improve patient outcomes, and enhance the quality of life for individuals living with AF.

## Figures and Tables

**Table 1:** Baseline characteristics of patients.

Characteristics of the Patients at Baseline	
Characteristic	Total Sample (N = 345)
Sex distribution	
Male—no. (%)	190 (55%)
Female—no. (%)	155 (45%)
Age distribution	
15–24 y.o.—no. (%)	7 (2%)
35–44 y.o.—no. (%)	7 (2%)
45–44 y.o.—no. (%)	3 (1%)
55–66 y.o.—no. (%)	62 (18%)
65–74 y.o.—no. (%)	97 (33%)
>75 y.o.—no. (%)	169 (49%)
Hospitalization days	
3–5 days—no. (%)	59 (17%)
6–10 days—no. (%)	241 (70%)
11–15 days—no. (%)	31 (9%)
>15 days—no. (%)	14 (4%)
Complications	
HF—no. (%)	110 (32%)
Stroke—no. (%)	59 (17%)
MI—no. (%)	17 (5%)
Risk factors/comorbidities	
HF—no. (%)	123 (35.6%)
HBP—no. (%)	186 (54%)
DM2—no. (%)	66 (19.13%)
Obesity—no. (%)	54 (15.65%)
LDL-c—no. (%)	14 (4%)
HTG—no. (%)	9 (2.6%)
TD—no. (%)	9 (2.6%)
LVF—no. (%)	204 (59%)
MR—no. (%)	207 (60%)
TR—no. (%)	90 (26%)
AR—no. (%)	66 (19%)
MS—no. (%)	21 (6%)
AS—no. (%)	38 (11%)
DM—no. (%)	110 (32%)
MI—no. (%)	17 (5%)
AH and/or AR—no. (%)	28 (8%)
HE—no. (%)	34 (10%)
PH—no. (%)	86 (25%)
COPD—no. (%)	38 (11%)
Other—no. (%)	21 (6%)
Labile INR—no. (%)	104 (30%)
Anticoagulant therapy before admission	
NOACs—no. (%)	252 (73%)
VKAs—no. (%)	55 (16%)
VKAs + aspirin—no. (%)	21 (6%)
Unspecified—no. (%)	17 (5)
During hospitalization	
LMWH—no. (%)	31 (9%)
LMWH + VKAs—no. (%)	93 (27%)
LMVH + VKAs + aspirin—no. (%)	21 (6%)
VKAs + aspirin—no. (%)	21 (6%)
NOACs—no. (%)	179 (52%)
After hospitalization	
NOACs—no. (%)	210 (61%)
VKAs + aspirin—no. (%)	114 (33%)
VKAs—no. (%)	21 (6%)

y.o.—years old, HF—heart failure, MI—myocardial infarction, HBP—high blood pressure, DM2—diabetes mellitus type 2, LDL-c—hypercholesterolemia, HTG—hypertriglyceridemia, TD—thyroid dysfunction, LVF—left ventricular failure, MR—mitral regurgitation, TR—tricuspid regurgitation, AR—aortic regurgitation, MS—mitral stenosis, AS—aortic stenosis, DM—dilated cardiomyopathy, AH and/or AR—abnormal hepatic and/or renal function, HE—hemorrhagic events, PH—pulmonary hypertension, COPD—chronic obstructive pulmonary disorder, NOACs—novel oral anticoagulants, VKAs—vitamin K antagonists, LMWH—low-molecular-weight heparin.

**Table 2:** CHA2DS2-VASc score.

Letter	Risk Factor	Score
C	Congestive heart failure/LV dysfunction	1
H	Hypertension	1
A_2_	Age ≥ 75	2
D	Diabetes mellitus	1
S_2_	Stroke/TIA/thromboembolism	2
V	Vascular disease	1
A	Age 65–74	1
S	Sex category (i.e., female sex)	1
	Maximum score	9

LV—left ventricular. TIA—transient ischemic attack.

**Table 3:** HAS-BLED score.

Letter	Risk Factor	Score
H	Hypertension	1
A	Abnormal renal and liver function (1 point each)	1 or 2
S	Stroke	1
B	Bleeding	1
L	Labile INRs	1
E	Elderly (e.g., age > 65 years)	1
D	Drugs or alcohol (1 point each)	1 or 2

## Data Availability

The data presented in this study are available on request from the corresponding authors. The data are not publicly available due to privacy reasons.
